# Infectious Disease Surveillance

**DOI:** 10.3201/eid1604.090584

**Published:** 2010-04

**Authors:** Marc A. Strassburg

**Affiliations:** Los Angeles County Department of Public Health, Los Angeles, California, USA; University of California, Davis, California, USA; TUI University, Cypress, California, USA

**Keywords:** Surveillance, Web, search engine, internet, infectious diseases, book review

This book devotes chapters to the usual infectious disease suspects and surveillance concepts and systems. I will not go into details of its contents and glowing attributes; the publisher (www.blackwellpublishing.com/book.asp?ref=9781405142663) and reviewers (*1*,*2*) have done a good job on this. Instead, I conducted a simple review to try and answer the following question: compared with a free Internet search, is purchase of a $195.00 book worth it? My hypothesis was that by entering the title of a chapter from the book in a search engine, one could find comparable content within the first 20 results (links) returned.

## Methods

After selecting the chapter that corresponded with every 50th page of the book, I visually scanned that chapter for scope and content. Then I performed a Google search using the chapter title and ranked the level of congruence between the topics in the book chapter and the topics at the Internet links as follows: fully comparable, >1 links covered most (≈%75) topics in the chapter; partially comparable, >1 links covered many (≈50%) of the topics; and not comparable, all links reviewed (up to 20) covered <50% of the topics. Each search result link was reviewed in successive order up to 20 results until >1 enabled a ranking of fully comparable. Links that led to other links would be followed only 1 level deep. Links that required log-in or payment or that reproduced a complete chapter were excluded.

## Results

Of the 40 chapters, 10 were reviewed. Of these, 4 were ranked as fully comparable, 4 as partially comparable, and 2 as not comparable.

For example, a rating of fully comparable was given for the chapter “Public Health Surveillance for Vaccine Adverse Events.” After 6 links were reviewed, 2 were found to provide similar information to that of the book chapter. A rating of partially comparable was given for “The Netherlands’ Infectious Diseases Surveillance Information System.” Of 20 links reviewed, several contained related information, but none were as complete as the book chapter and some provided a more global view. A rating of not comparable was given for “Use of Molecular Epidemiology in Infectious Disease Surveillance” after a review of all 20 links. Although several links provided information on the topic, none provided as organized and clear a summary as did the book chapter.

## Discussion

Extrapolation of these findings to all 40 chapters is difficult because of variation in topics and titles, and extrapolation to other research areas may depend on the proportion of information published without restrictions, i.e., without requesting registration, passwords, or money. Overall, a considerable amount of Internet information was obtained for most chapters evaluated, although usually >1 link had to be reviewed. The convenience of being able to read at one’s own pace a book containing the full gamut and organized presentation of such subject matter must be weighed against the timeliness and cost differential of an Internet search.

## Conclusions

This book is a good value for public health students and professionals frequently involved in surveillance and surveillance issues. It is not necessarily a good value for those with only infrequent needs to find information quickly or to find up-to-date information on several surveillance topics.

## References

[1] DeMaria A Jr. Infectious disease surveillance. N Engl J Med. 2008;358:1413–4. 10.1056/NEJMbkrev59336

[2] Grassly NC. Infectious disease surveillance. JAMA. 2008;300:1591–2. 10.1001/jama.300.13.1591-b

